# Special Issue “Recent Advances in Morbillivirus Vaccine Development and Oncolytic Virotherapy”

**DOI:** 10.3390/v12030341

**Published:** 2020-03-20

**Authors:** Liang-Tzung Lin

**Affiliations:** 1 Department of Microbiology and Immunology, School of Medicine, College of Medicine, Taipei Medical University, Taipei 110, Taiwan; ltlin@tmu.edu.tw; Tel.: +886-2-2736-1661 (ext. 3911); 2 Graduate Institute of Medical Sciences, College of Medicine, Taipei Medical University, Taipei 110, Taiwan

Members of the *Morbillivirus* genus are enveloped, negative-strand RNA viruses that include a number of highly contagious pathogens important to humans and animals. They are known to be transmitted via the respiratory route and cause febrile diseases that can be fatal. Despite the availability of attenuated vaccines against several members, these viruses remain responsible for significant morbidity and mortality in their natural hosts worldwide. The development of molecular biology techniques over the past decades has helped increase the understanding of morbillivirus pathogenesis and explore the possibility to engineer their genomes as viral vectors. This Special Issue of *Viruses* explores recent advances in recombinant morbilliviruses platforms, especially measles virus (MV) and canine distemper virus (CDV), for novel vaccine development and oncolytic virotherapy against cancers. Topics in this special issue include parameters involved during the viral vector production, strategies of viral vector engineering, and the underlying mechanisms of the therapeutic effects exhibited by these vectors.

Loewe et al. [1] performed a forced degradation study to identify potential factors that could affect the titer of MV produced during the downstream processing of therapeutic viral vectors. They report that MV is highly sensitive to thermal stress and acidic media, while freeze-thawing, buffer types, osmolality and ionic strength do not affect the stability of MV in general. Ammour et al. [2] examined the oncolytic activity of a vaccine strain MV Leningrad-16 (L-16) in metastatic melanoma cell lines in vitro and in vivo. Scheubeck et al. [3] demonstrated that starvation could sensitize human colorectal cancer cells to oncolytic MV treatment, and that this effect is minimal on normal colon cells. Backhaus et al. [4] developed an interleukin-15 (IL-15)-armed MV vector and compared its oncolytic profile with a previously generated IL-12-armed vector in mouse models. While IL-15 expression increased T and NK cell infiltrations, IL-12 enhanced the viral gene expression, as well as immune activation, and displayed higher efficacy. Busch et al. [5] attempted to design recombinant MV vectors that enhance the activation of antigen-specific CD8+ T cells. They generated MV encoding melanoma antigen tyrosinase-related protein-2 (TRP-2) or ovalbumin (OVA) and report increased interferon-γ secretion from CD8+ T cells or dendritic cells, which is indicative of cytotoxic T-cell response, after co-culturing with the infected target cells. On the other hand, Armando et al. [6], on the other hand, investigated the impact of CDV infection on angiogenesis in a canine histiocytic sarcoma cell line. Persistent CDV infection in this cell line resulted in the downregulation of hypoxia-inducible factor (HIF)-1α angiogenic pathway, suggesting another potential anti-cancer activity of CDV besides direct oncolysis. Finally, Zhao et al. [7] contribute a comprehensive review on the development of recombinant CDV vectors using reverse genetic system, and discussed their use as animal vaccines or oncolytic agents for human and animal cancers. 

With the above studies, we hope that this Special Issue will provide further insights into the development of vaccine and therapeutic applications using viral vectors in the morbillivirus family.
